# Job Disengagement Among Physical Education Teachers: Insights From a Cross-sectional Web-Based Survey With Path Modeling Analysis

**DOI:** 10.2196/29130

**Published:** 2022-12-01

**Authors:** Nasr Chalghaf, Wen Chen, Amayra Tannoubi, Noomen Guelmami, Luca Puce, Noureddine Ben Said, Maher Ben Khalifa, Fairouz Azaiez, Nicola Luigi Bragazzi

**Affiliations:** 1 Higher Institute of Sport and Physical Education of Gafsa, University of Gafsa Gafsa Tunisia; 2 Department of Child Psychology, The Children's Hospital, Zhejiang University School of Medicine, National Clinical Research Center for Child Health, National Children's Regional Medical Center Hangzhou, Zhejiang China; 3 Higher Institute of Sport and Physical Education of Kef, University of Jendouba Jendouba Tunisia; 4 Department of Neuroscience, Rehabilitation, Ophthalmology, Genetics, Maternal and Child Health (DINOGMI), University of Genoa Genoa Italy; 5 Department of Biomechanics and Motor Behavior, College of Sport Sciences and Physical Activity, King Saud University Ryadh Saudi Arabia; 6 Department of Human Sciences, Higher Institute of Sport and Physical Education of Sfax, University of Sfax Sfax Tunisia; 7 Laboratory for Industrial and Applied Mathematics (LIAM) Department of Mathematics and Statistics York University Toronto, ON Canada

**Keywords:** Work Disengagement Scale, work, job, job satisfaction, family–work conflict, perceived stress, physical education, PLS-SEM, SmartPLS, teacher, engagement, Arab, stress, primary school, secondary school, development, measurement, scale, tool, fitness, teacher, educator, school, satisfaction, digital tool, mental health, family, cross-sectional, survey, modelling, psychology

## Abstract

**Background:**

Physical education teachers often experience stress and job disengagement.

**Objective:**

This study’s aims were as follows: (1) to adapt in the Arabic language and test the reliability and the validity of the work–family conflict (WFC) and family–work conflict (FWC) scales, (2) to develop and assess the psychometric properties of work disengagement among physical education teachers, and (3) to evaluate an explanatory model by presenting the mediating role of perceived stress as a major influencing factor in work disengagement and job satisfaction.

**Methods:**

A total of 303 primary and secondary school physical education teachers, comprising 165 (54.5%) men and 138 (45.5%) women participated voluntarily in our study. The measuring instruments are the Work Disengagement Scale, the Perceived Stress Scale, the WFC scale, the FWC scale, and the 9-item Teacher of Physical Education Job Satisfaction Inventory.

**Results:**

The Arabic language versions of the WFC and FWC scales had reasonably adequate psychometric properties, which were justified by confirmatory factor analyses and by the measurement of reliability, convergent, and discriminant validity through the measurement model using SmartPLS software. Similarly, the structural model established with SmartPLS confirmed strong links of the concepts of FWC, WFC, the job satisfaction questionnaire, and perceived stress with work disengagement among teachers of physical education.

**Conclusions:**

There is a growing interest in helping teachers cope with the daily pressures of work and family. A positive organizational context is a context with clear values regarding work priorities, which constitutes the basis of a feeling of shared responsibility and professional support. Good conditions can act as protective factors reducing work stress and positively influencing personal well-being, work attitudes, work commitment, and professional efficiency. Additional research on teachers is needed to examine the relationship between perceived work stress and the role of families, along with the extent to which this association can have a significant impact on teachers’ commitment to work.

## Introduction

The problem of perceived stress has a great impact on the psychological health and well-being of employees, and it is always a very important factor to study [1].

Perceived stressors have been associated with a wide range of both mental and physical health issues, including depression and anxiety disorders, suicide, workplace accidents and injuries, and cardiovascular risk. Researchers generally agree that stress is a serious problem in many countries and has a very negative impact in the professional world. Contemporary organizations have opted for individual and organizational stress management [2]. However, it is frequently reported that the majority of stress management interventions are ineffective [3] and lead to disengagement from work. Other psychosocial factors, such as work–family conflict (WFC) and family–work conflict (FWC), can also increase stress. WFC can occur when demands from one role at home can affect one’s ability to meet the demands associated with another role at the workplace. The reverse is known as FWC, with issues at work clashing with family responsibilities and duties. FWC refers to “a form of inter-role conflict in which the general demands, the time spent, and the tensions created by the family interfere with the execution of work-related responsibilities” [4].

The features of a successful teacher include personal characteristics, topic knowledge, and highly qualified teaching skills, all of which can have an impact on pupils [5]. Looking into how students perceive their physical education teachers’ skill sets may help improve academic quality and boost participation in both extracurricular sports and physical education programs. This strategy for increasing physical activity has been developed as a result of the discovery that students’ perceptions of the school setting were in fact associated with their well-being, academic success, and positive attitudes toward school-based physical exercises. The physical education teacher, as physical activity specialist, should give a positive primary representation of this environment and should act as a role model for the pupils in terms of preserving physical health and leading an active life. Teachers’ influences are much more nuanced than merely imparting knowledge and transferring abilities. Recent social and economic developments have prompted researchers to look for new strategies for enhancing teachers’ professionalism, given that, in recent years, the condition of physical education teachers has changed because of the introduction of new programs and stringent quality checks: perceived stress and professional disengagement linked to the profession among many teachers has been increasing and has become dramatically remarkable [1].

It is, therefore, very important to focus on the professional disengagement linked to the professional stress among physical education teachers and to highlight 4 factors that are likely to increase it: relations with superiors, the professional environment, the family circle, and job satisfaction. In this regard, relationships and interactions with colleagues and students sometimes create a critical organizational environment and are, therefore, often potential sources of stress. Good interpersonal relationships help achieve personal goals among individuals and organizational goals of whole teams, while poor interpersonal relationships cause stress and affect teachers’ performance and well-being.

In Arab countries including Tunisia, we lack valid and reliable tools to measure concepts such as WFC, FWC, and work engagement. Therefore, to test relational models, it is necessary to adapt or validate measurement scales in Arabic.

The objective of this study was to develop a measurement scale of job disengagement among physical education teachers to verify the psychometric properties of WFC and FWC and to present an explanatory model describing the mediating role of perceived stress and job satisfaction, along with the relationships between family and work as an indirect effect.

## Methods

### Participants

We used a snowball sampling procedure to collect cross-sectional data for physical education teachers in Tunisia. Overall, 303 physical education teachers with a mean age of 36.46 (SD 7.92) years participated in the study. The sample comprised 165 (54.5%) males and 138 (45.5%) females. Furthermore, 162 (53.5%) of them were physical education teachers at primary school and 141 (46.5%) of them taught at secondary school. All teachers have more than 15 years of experience in their professional careers.

### Ethics Approval

The study protocol received ethical clearance from the United Nations Educational, Scientific and Cultural Organization chair “Health Anthropology Biosphere and Healing Systems”; University of Genoa, Genoa, Italy; the Higher Institute of Sport and Physical Education of Sfax, Sfax, Tunisia; the Faculty of Letters and Human Sciences of Sfax, Sfax; and the Higher Institute of Sport and Physical Education of Kef, Kef, Tunisia. The study was approved by the ethical committee of the University of Sfax (020/2021).

All study participants provided written informed consent. Teachers were extensively informed about the purposes and procedure of the study and were advised that the results would be made available to them upon completion of the study only in aggregate form, with no possibility to trace back to individual teachers’ scores, thus ensuring anonymity and preserving the privacy of each participant.

This study was carried out following the ethical principles of the 1964 Helsinki declaration and its subsequent amendments.

### Instruments

#### Arabic Version of the Perceived Stress Scale

The 10-item Perceived Stress Scale [6] measures global perceived stress experienced across the past 30 days, on a 5-point scale (0=“never,” 1=“almost never,” 2=“once in a while,” 3=“often,” and 4=“very often”). The validity and reliability of the Arabic version of the Perceived Stress Scale were assessed with acceptable results. The overall Cronbach α coefficient was .80 for the Arabic version of the Perceived Stress Scale. The test-retest reliability had an intraclass correlation coefficient of .90 [7].

#### WFC and FWC

We used 5 items of 2 Arabic versions of the WFC and FWC scales [4]. The psychometric properties of the 2 original instruments were satisfactory and tested on 3 different samples. The reference model shows an adequate fit and factor invariance across the goodness-of-fit index (GFI) and the comparative fit and Tucker-Lewis indices, which are in the range of .90 and above. Responses were rated on a 5-point Likert scale ranging from 1=“strongly disagree” to 5=“strongly agree.”

A discussion group was organized with experts of the Arabic language, sports, and physical education, as well as the humanities and applied sciences to translate the items of the 2 tools. Then, several corrections made it possible to reformulate the questions that seemed unclear. This has improved the tool. Finally, a pilot study was carried out to test the preliminary properties and the readability of the questionnaires—this test confirmed the validity of this work.

#### Job Dissatisfaction

Job dissatisfaction was assessed with an inverse score based on a 5-point Likert scale—the Arabic version of the 9-item Teacher of Physical Education Job Satisfaction Inventory (TPEJSI-9) [8].

Internal consistency α coefficients of the TPEJSI were all >.80: for satisfaction with colleagues, α=.87; for satisfaction with parents, α=.87; and for satisfaction with students, α=.86. The tool also provides good exploratory factor analysis factor loadings, acceptable confirmatory factor analysis (CFA) fit indices, and excellent convergent validity.

#### The Work Disengagement Scale

The Work Disengagement Scale (WDES) was developed in the Arabic language from the Utrecht Work Engagement Scale validated by Schaufeli* *et al [9]. This scale contains 9 items that present disengagement from work on a 6-point Likert scale.

### Procedure

The first step of the validation process was the translation of the original English versions of the WFC and FWC scales to classical Arabic by a committee.

After informed consent was obtained, the selected participants answered a paper version structured questionnaire that included all scales (Multimedia Appendix 1). The entire procedure of questionnaire administration lasted over 2 months. A proper time period (approximately 60 minutes) was ensured for each participant to answer the questionnaire thoroughly.

### Statistical Analysis

Analyses were performed in SPSS Statistics software (version 22.0; IBM Corp) and SmartPLS (version 3.2.9; SmartPLS GmbH).

Before commencing any statistical analysis, data were visually inspected for potential outliers. The normality of data distribution was checked using the Pearson-D’Agostino omnibus test. Means and SDs for ordinal data were computed for the entire sample.

Questionnaires’ scores were also checked for skewness and kurtosis, computing the Mardia multivariate skewness and kurtosis statistics [10].

CFA was used to verify the psychometric properties of the WFC and FWC scales and the WDES. Several fit indices were evaluated to determine model fit.

When the model was tested using CFA, as suggested and recommended by many scholars, a wide range of fit indices was calculated and reported, including the following: (1) discrepancy indices (including the chi-square and the Steiger and Lind [11] root mean square error of approximation), (2) tests comparing the target model with the null model (including the Bentler and Bonett [12] normed fit index; the Bentler and Bonett [12] nonnormed fit index, known also as the Tucker-Lewis index; the Bentler comparative fit index; and the James-Mulaik-Brett parsimony GFI [13]), and (3) information theory goodness-of-fit measures (the Joreskog GFI and the Joreskog adjusted GFI).

For the estimation of the model, we used the SmartPLS software (version 3.2.9) to analyze the path model. The PLS method uses least squares regression techniques to estimate the models. The objective of PLS modeling is to maximize the explained variance of the dependent latent variable, whereas that of covariance-based methods is to reproduce the theoretical covariance matrix.

**Figure 1:**
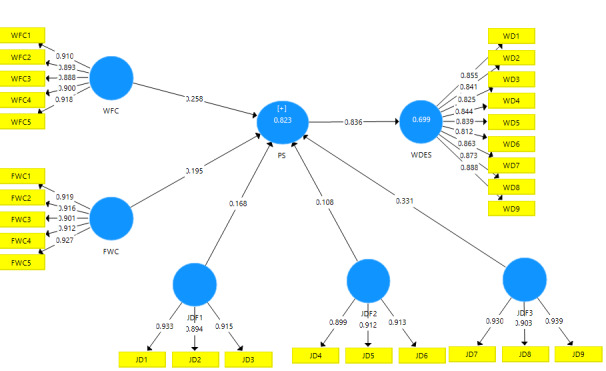
SmartPLS model. FWC: family–work conflict; JD: job dissatisfaction; PS: perceived stress; WD: work disengagement; WDES: Work Disengagement Scale; WFC: work–family conflict.

**Figure 2:**
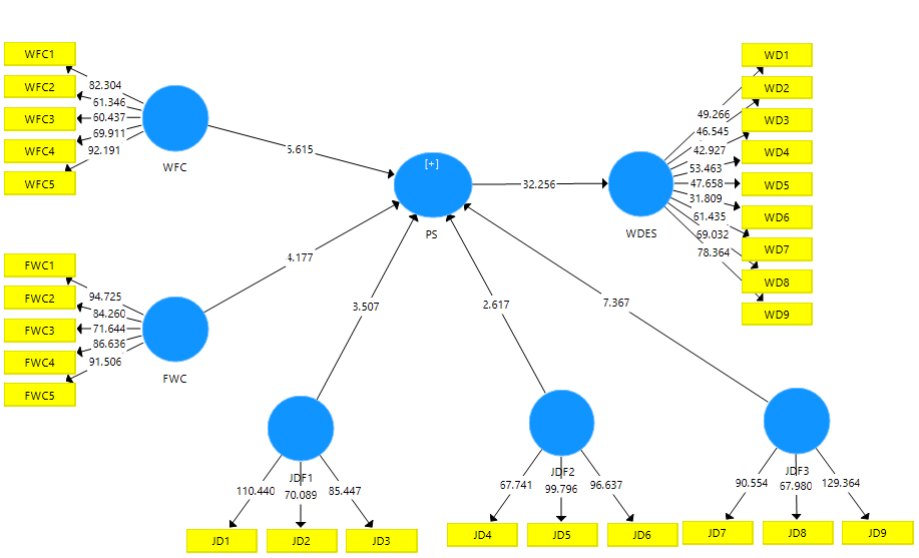
SmartPLS bootstrapping model. FWC: family–work conflict; JD: job dissatisfaction; PS: perceived stress; WD: work disengagement; WDES: Work Disengagement Scale; WFC: work–family conflict.

The measurement model defines the relationships between the latent and observable variables. It contains indications of the operationalization of the theoretical concepts of a study. The need to evaluate the measurement model is an essential need since the relationships between the latent variables and the theoretical concept or among the different concepts may be inaccurate. For the different measurement scales, the reflective model was adopted following bibliographic recommendations. In a reflective measurement scale, the direction of causality is from the latent variable to the indicators (represented in blue and yellow, respectively, in Figures 1 and 2). If the indicators are highly correlated and interchangeable, they are reflective and their reliability and validity must be thoroughly examined [14-16].

## Results

### Overview

We started with the factorial examination of the 2 adapted scales—WFC and FWC scales—as well as the constructed WDES (Table 1).

The Cronbach α coefficients were remarkable for the 3 scales: Cronbach α values were .943, .952, and .952 for the WFC scale, FWC scale, and WDES.

**Table 1:** Confirmatory factor analysis fit index for the work–family conflict scale, family–work conflict scale, and Work Disengagement Scale.

Scales	Chi-square	*df*	Chi-square / *df*	Normed fit index	Goodness-of-fit index	Adjusted goodness-of-fit index	Tucker-Lewis index	Comparative fit index	Root mean residual	Root mean square error
Work–family conflict scale	3.47	5	0.70	.99	.99	.98	1	1	.02	0
Family–work conflict scale	7.41	5	1.48	.995	.99	.97	.997	.998	.020	.040
Work Disengagement Scale	59.62	27	2.21	.97	.96	.93	.98	.98	.03	.063

### Evaluation of the Measurement Model

The findings of the SmartPLS algorithm are pictorially shown in Figure 1.

Hair et al [17] argue that to verify the measurement model, it is necessary to verify its reliability using composite reliability (CR) and its validity by measuring convergent validity and discriminant validity. According to Fornell and Larcker [18], the CR must be ≥.6 to have a reliable construct. Here, we obtained values of .963, .939, .934, .946, .923, .959, and .956 for the variables “FWC,” “JDF1,” “JDF2,” “JDF3,” “PS,” “WDES,” and “WFC” respectively (Table 2). These values are well above the recommended threshold. This indicates that our measurement model is reliable.

**Table 2:** Reliability and average variance extracted (AVE).

Variables	Cronbach α	ρ_A_	Composite reliability	AVE
Family–work conflict	.952	.952	.963	.838
Job dissatisfaction (F1)	.902	.904	.939	.836
Job dissatisfaction (F2)	.893	.894	.934	.824
Job dissatisfaction (F3)	.914	.917	.946	.854
Perceived stress	.907	.907	.923	.545
Work disengagement	.952	.952	.959	.721
Work–family conflict	.943	.943	.956	.814

Convergent validity is measured using the loading factor, which must be >.6 [19] and the average variance extracted (AVE), which must be >.5 [18]. Our results show that all measures—Cronbach α, ρ_A_, and CR—of the variables are >.7 and the AVE is >.5 (Table 2). These values respect the recommendations of the authors, which indicates that the measures of each variable of the converging model allow us to properly represent the variable in question.

The discriminant validity of a construct can be assessed by comparing the square root of the values of the AVE with correlations of latent variables [18]. The square roots of the AVE coefficients are presented in the correlation matrix along the diagonal. The square root of the AVE of each construct must be greater than its strongest correlation with any other construct to demonstrate discriminant validity [15].

Table 3 shows that the square root of the AVE of each variable is greater than its correlation coefficients with other variables. These results indicate that the measurement model has good discriminant validity. Based on the results of CR, convergent validity, and discriminant validity, we can thus deduce that our measurement model is reliable and valid. Hence, after evaluating the measurement model, we will now evaluate our structural model.

**Table 3:** Reliability and average variance extracted.

Variable	PS^a^	FWC^b^	JD^c^F1	JDF2	JDF3	WDES^d^	WFC^e^
PS	.738	—^f^	—	—	—	—	—
FWC	.794	.915	—	—	—	—	—
JDF1	.752	.675	.914	—	—	—	—
JDF2	.684	.585	.644	.908	—	—	—
JDF3	.799	.642	.679	.614	.924	—	—
WDES	.836	.691	.644	.577	.670	.849	—
WFC	.792	.815	.614	.586	.631	.677	.902

^a^PS: perceived stress.

^b^FWC: family–work conflict.

^c^JD: job dissatisfaction.

^d^WDES: Work Disengagement Scale.

^e^WFC: work–family conflict.

^f^Not applicable.

### Assessment of the Structural Model

Evaluation of the structural model was carried out by calculating the path coefficients to evaluate the hypotheses, the coefficient of determination (*R*²), the effect size (f²), and the predictive relevance (Q²) [17,20].

*R*² represents the explanation’s power of the dependent variable by the independent variables. Falk and Miller [21] propose .10 as the minimum value of *R*² for it to be accepted. The results of our model show that the *R*² of the variables perceived stress (PS) and WDES are equal to .823 and .699, respectively. This indicates that the influence of job dissatisfaction (JD), WFC, and FWC represents 82% of the variance of PS. Similarly, the influence of all these variables explains 70% of the variance in work disengagement.

The f² represents the influence of each independent variable on the dependent variable. According to Cohen [22], (1) f²>.35 implies that the independent variable has a large effect on the dependent variable, (2) .15<f²<.35 implies that the independent variable has a medium effect on the dependent variable, and (3) .02<f²<.15 implies that the independent variable has a small effect on the dependent variable. Our results show that PS has an f² of 2.32, whereas the f² for WFC, FWC, JDF1, JDF2, and JDF3 are .12, .07, .06, .03, and .27, respectively, and this has a small effect.

The predictive relevance Q² represents the predictive capacity of the model in measuring endogenous variables. Using the blindfolding method, we found that the Q² is equal to .44 and .50 for the variables PS and WDES, respectively. All these values are positive, which indicates that the model has good predictive capacity [17,23], which suggests that the Q² is greater than 0. Hypothesis testing is shown in Table 4.

**Table 4:** Hypothesis testing.

	Path coefficient	SD	*t*-value	*P* value	Hypothesis
FWC^a^→PS^b^	0.195	0.047	4.177	<.001	Confirmed
FWC→WDES^c^	0.163	0.039	4.161	<.001	Confirmed
JD^d^F1→PS	0.167	0.048	3.507	<.001	Confirmed
JDF1→WDES	0.140	0.041	3.455	.001	Confirmed
JDF2→PS	0.107	0.041	2.617	.009	Confirmed
JDF2→WDES	0.089	0.035	2.581	.01	Confirmed
JDF3→PS	0.330	0.045	7.367	<.001	Confirmed
JDF3→WDES	0.276	0.039	7.151	<.001	Confirmed
PS→WDES	0.837	0.026	32.256	<.001	Confirmed
WFC^e^→PS	0.260	0.046	5.615	<.001	Confirmed
WFC–WDES	0.217	0.038	5.641	<.001	Confirmed

^a^FWC: family–work conflict.

^b^PS: perceived stress.

^c^WDES: Work Disengagement Scale.

^d^JD: job dissatisfaction.

^e^WFC: work–family conflict.

## Discussion

### Principal Findings

Our study aimed to (1) adapt in Arabic language and examine the reliability and the validity of the WFC and FWC scales, (2) design and analyze the psychometric properties of the work disengagement among physical education instructors, and (3) explore an explanatory model by demonstrating the mediation function of perceived stress as a significant influencing factor in work disengagement and job satisfaction.

The Arabic versions of the WFC and FWC scales had reasonably adequate psychometric properties, which were justified by CFA and the measure of reliability, convergent, and discriminant validity. Similarly, the developed tool WDES showed good reliability and adequate fit indices on CFA. These results of the WDES have been supported by measurement model review established in SmartPLS.

The structural model established with SmartPLS software confirmed strong links between PS and FWC, WFC, and job satisfaction among physical education teachers. These results are in line with those of Caesens [24], which revealed relationships among perceived organizational support, job satisfaction, and PS.

In line with our results, the study by Watson et al [25] conducted with 53 beginning teachers, revealed through regression analysis that holistic well-being and PS contributed significantly to the variance in job satisfaction. Furthermore, Orgambídez-Ramos et al [26] revealed by hierarchical models that job satisfaction was significantly predicted by stress and work engagement.

Besides, perceived work stress has been found to mitigate the effect of high job demands on WFC [27]. Furthermore, significant gender differences were found in PS levels, FWCs, and commitment to work.

In another study by Gandhi et al [28] performed with 150 nurses (both male and female), the results showed that PS and perceived job satisfaction were negatively correlated. These results confirmed those of Guppy and Gutteridge [29], who found that job satisfaction was negatively linked to stress.

Among physical education teachers, Koustelios and Tsigilis [30] examined the multivariate relationship between job satisfaction and burnout as an extremely stressful situation experienced by Greek physical education teachers in school. Canonical correlation analysis revealed a negative multivariate relationship between the two constructs (*R*_c_=.61).

To explain these findings, Laugaa et al [31] claimed that teaching working conditions have been deteriorating. Various researchers in Quebec, Canada, have also identified several sources of stress among teachers: workload (source most often noted) [32], lack of time [33], resources [32], and recognition or respect [33]. However, these studies have not examined relationships with families.

Furthermore, a certain degree of stress can sometimes have positive effects such as learning [34], hope, joy, passion, or satisfaction [33]; however, prolonged stress among teachers can have many negative consequences [35]: early retirement [32], effects on family life and relationships [33], and effects on satisfaction (the most stressed individuals are also the least satisfied ones [35]). Similarly, Chu [36] studied the influence of PS on WFC and mental health. The results show that PS is an effective predictor of WFC and mental health.

### Limitations

This research has a number of limitations. First, the sample size did not allow a confirmatory model to be generated using the structural equation modeling approach instead of the partial least square predictive technique. Second, other factors that are related to work engagement such as grit and personality traits of study participants were not examined. Third, moderating effects such as expertise and gender were not examined. It is recommended that these moderating effects should be assessed in further work.

### Conclusions

The results of the reliability and CFA suggest that the WFC scale, FWC scale, and WDES are valid and reliable and can measure all 3 concepts in a Tunisian context. These results were supported by the SmartPLS model. In addition, the structural model’s results support stress as a major influencing factor for work disengagement in physical education teachers. This model also shows that this PS stems from bidirectional FWC.

Therefore, there is growing interest in helping teachers cope with the daily pressures at work and family. A positive organizational context is a context with clear values regarding work priorities, which constitutes the basis of a feeling of shared responsibility and professional support.

Good conditions can act as protective factors reducing work stress and positively influencing personal well-being, work attitudes, work commitment, and professional efficiency. Additional research is needed to examine the relationship between perceived work stress and the role of teachers’ families and the extent to which this association can have a significant impact on teachers’ commitment to work.

## Appendix

Multimedia Appendix 1 Scales in Arabic.

## Abbreviations

AVE: average variance extracted
CFA: confirmatory factor analysis
CFI: comparative fit index
CR: composite reliability
FWC: family–work conflict
GFI: goodness-of-fit index
JD: job dissatisfaction
PS: perceived stress
TPEJSI-9: 9-item Teacher of Physical Education Job Satisfaction Inventory (
WDES: Work Disengagement Scale
WFC: work–family conflict
