# Supplementary material for: Job Disengagement Among Physical Education Teachers: Insights From a Cross-sectional Web-Based Survey With Path Modeling Analysis
Source: JMIR Form Res. 2022 Dec 1;6(12):e29130. doi: 10.2196/29130 (PMC9756116; doi:10.2196/29130)
Supplement: Multimedia Appendix 1 [file formative_v6i12e29130_app1.docx]

**Multimedia Appendix 1.** Scales in Arabic.

| **مقياس نزاع بين العمل والأسرة** |
| --- |
| 1. متطلبات عملي تتعارض مع متطلباتمنزلي وحياتي العائلية. |
| 2. مقدار الوقت الذي أقضيه في وظيفتي يجعل من الصعب عليّ الوفاء بالمسؤوليات الأسرية. |
| 3. الأشياء التي أريد القيام بها في المنزل لا يتم إنجازها بسبب المطالب التي يفرضها عليّ عملي. |
| 4. ينتج عن عملي إجهادًا يجعل من الصعب أداء الواجبات الأسرية. |
| . 5. بسبب الواجبات المتعلقة بالعمل ، لا بد لي من إجراء تغييرات على خططي للأنشطة العائلية. |
| **مقياس نزاع العمل الأسري** |
| 1. تتعارض مطالب عائلتي أو زوج)ت(ي / شريك)ت(ي مع الأنشطة المتعلقة بالعمل. |
| 2. لا بد لي من تأجيل القيام بالأشياء في العمل بسبب الطلبات في المنزل. |
| 3. الأشياء التي أريد القيام بها في العمل لا يتم إنجازها بسبب مطالب عائلتي أو زوجتي / شريكي. |
| 4. تتعارض حياتي المنزلية مع مسؤولياتي في العمل مثل الذهاب إلى العمل في الوقت المحدد ، وإنجاز المهام اليومية ، والعمل متأخر , بعد فوات الوقت. |
| 5. الإجهاد المرتبط بالأسرة يتعارض مع قدرتي على أداء الواجبات المتعلقة بالوظيفة. |
| **إستبيان عدم الإلتزام** في العمل |
| 1. عندما أقوم بعملي ، أشعر بالتعب. |
| 1. أشعر بالإرهاق والعجز عندما أذهب إلى العمل.. |
| 1. أنا غير مبال باالأنشطة المتعلقة بالعمل. |
| 1. عملي لا يلهمني. |
| 1. عندما أستيقظ في الصباح، أفضل عدم الذهاب إلى العمل. |
| 1. أشعر بالحزن عندما أعمل بشكل مكثف. |
| 1. لست فخورًا بعملي. |
| 1. عملي لا يهمني. |
| 1. أشعر بالتشتت عندما أعمل. |
